# Clinical Potential of Dental Pulp Stem Cells in Pulp Regeneration: Current Endodontic Progress and Future Perspectives

**DOI:** 10.3389/fcell.2022.857066

**Published:** 2022-04-11

**Authors:** Kyu Hwan Kwack, Hyeon-Woo Lee

**Affiliations:** ^1^ Department of Dentistry, Graduate School, Kyung Hee University, Seoul, South Korea; ^2^ Department of Pharmacology, School of Dentistry, Graduate School, Institute of Oral Biology, Kyung Hee University, Seoul, South Korea

**Keywords:** regeneration medicine, dental pulp stem cells, dental pulp regeneration, immunomodulation, allogeneic transplantation, mesenchymal stem cells

## Abstract

Dental caries is a common disease that not only destroys the rigid structure of the teeth but also causes pulp necrosis in severe cases. Once pulp necrosis has occurred, the most common treatment is to remove the damaged pulp tissue, leading to a loss of tooth vitality and increased tooth fragility. Dental pulp stem cells (DPSCs) isolated from pulp tissue exhibit mesenchymal stem cell-like characteristics and are considered ideal candidates for regenerating damaged dental pulp tissue owing to their multipotency, high proliferation rate, and viability after cryopreservation. Importantly, DPSCs do not elicit an allogeneic immune response because they are non-immunogenic and exhibit potent immunosuppressive properties. Here, we provide an up-to-date review of the clinical applicability and potential of DPSCs, as well as emerging trends in the regeneration of damaged pulp tissue. In addition, we suggest the possibility of using DPSCs as a resource for allogeneic transplantation and provide a perspective for their clinical application in pulp regeneration.

## Introduction

Dental pulp is a tissue in the center part of the tooth, surrounded by dentin, and plays a crucial role in maintaining the vitality of teeth by supplying essential factors through the apical foramen. The neural network distributed in the pulp tissue through the apical foramen plays a role in protecting the teeth by recognizing harmful stimuli, and the blood vessels in the pulp tissue supply nutrients to the teeth and remove waste products. Dental pulp has high functional regenerative capacity as it is responsible for the maintenance as well as the repair of periodontal tissue in response to various types of damage. Dental pulp cells proliferate when periodontal tissue is damaged, migrate to the damaged site, then differentiate into odontoblasts to form reparative dentin (Tziafas et al., 2000; Dimitrova-Nakov et al., 2014). Dental caries is one of the most prevalent diseases worldwide and has maintained its prevalence and incidence over the past two decades (Kassebaum et al., 2015). According to the most recent epidemiological data, the overall prevalence of total caries among youth aged in the United States is 45.8% (Fleming and Afful, 2018). There are no symptoms in the initial stages of caries, and symptoms begin only when the carious lesion grows and progresses to the dentin (Selwitz et al., 2007). When dental caries progresses and an inflammatory reaction occurs in the dental pulp, pulp tissue ischemia with severe pain occurs. The current common clinical treatment involves removing the damaged dental pulp tissue, disinfecting it, and then filling it with artificial fillings (Morotomi et al., 2019). Although initial root canal treatment (RCT) has a high success rate and a predictable prognosis after treatment, the possibility of reinfection still exists (Salehrabi and Rotstein, 2004; Torabinejad et al., 2007; de Chevigny et al., 2008). Even if the treatment is successful, the vitality of the pulp is lost, and the perception and immune function completely disappear, reducing the resistance to external stimuli and weakening the teeth (Miran et al., 2016). Moreover, when reinfection occurs and retreatment is performed, the success rate decreases, and repeated RCT makes the teeth more fragile and prone to cracking or even fracture of the roots (Van Nieuwenhuysen et al., 1994; Imura et al., 2007; Ng et al., 2008). To solve these problems, a tissue regeneration approach that replaces damaged pulp with healthy pulp is an ideal treatment option. Therefore, novel strategies for regenerating functional pulp are essential as pulp stem cells are emerging as promising candidates.

Mesenchymal stem cells (MSCs) are multipotent cells that can differentiate into all types of cells derived from the three embryonic germ layers, including osteoblasts, chondroblasts, and adipocytes. MSCs have been found in various tissues, such as bone marrow, adipose tissue, and dental tissues (Pittenger et al., 1999; Bianco et al., 2001). Because of these characteristics, MSCs have been recognized as a promising source of stem cells in regenerative therapy. However, their application has limitations such as safety and accessibility issues; thus, MSC-like cells isolated from dental tissues have begun to attract attention. MSC-like cells isolated from dental tissues have the advantage of being easily accessible from extracted teeth or from periodontal tissues that come with the extracted teeth (Gronthos et al., 2000; Gronthos et al., 2002; Miura et al., 2003). In addition, they can differentiate into nerves and blood vessels, which are necessary structures constituting the pulp tissue, and can be cryopreserved to store cells. Importantly, they may not elicit an immune response in allogeneic transplantations because they are non-immunogenic and have strong immunosuppressive properties (Kwack et al., 2017). With these advantages, DPSCs are the most important source of stem cells for pulp tissue regeneration because they exist in the original dental pulp and are prone to repair the damaged dental pulp (Zhang et al., 2017). In this review, we focus on DPSCs and provide an up-to-date review of their potential, including their immunosuppressive properties. In addition, the latest clinical methods for the regeneration of pulp tissue and the clinical applicability/strategies of DPSCs have also been discussed.

## Dental Caries/Pulpitis

Dental caries is a common disease worldwide; however, it shows no symptoms until certain degree of progression. Untreated caries in permanent teeth are the most prevalent worldwide, and the prevalence and incidence of dental caries has been steadily maintained (Kassebaum et al., 2015). Dental caries is caused by complex interactions between the dietary supply of fermentable carbohydrates, acid-producing bacteria, and many host factors, including saliva and teeth (Selwitz et al., 2007). As a result of these interactions, the bacteria form a biofilm and cause demineralization of the outermost hard part of the tooth, the enamel, by acidogenic byproducts of bacterial metabolism. As enamel demineralization continues, dentin is exposed to bacterial invasion, resulting in further demineralization and cavitation. If caries progresses untreated, they turn to deep caries that penetrate the entire thickness of the dentin with specific pulp exposure. When dental pulp cells are exposed to dental caries, they respond directly by expressing various chemokines and cytokines to promote cellular defense processes and attempt repair (Farges et al., 2015). Fibroblasts, the principal cells of the pulp, can secrete factors important for the recruitment of stem cells, and the recruited stem cells are directly involved in repair (Frozoni et al., 2012; Jeanneau et al., 2017). In addition, bone marrow fibrocytes play a role in early wound healing by migrating to the damaged pulp site (Yoshiba et al., 2018). Several progenitor cell populations, including DPSCs, migrate to injured pulp sites and differentiate into odontoblast-like cells during reparative dentinogenesis (Gronthos et al., 2002). To undergo such a pulpal repair process, low-grade inflammation must progress to stimulate the regenerative response (Cooper et al., 2010). If inflammation is not removed despite a series of repair processes, it will eventually lead to an inflammatory pulpal reaction, resulting in pulp necrosis and abscesses (Reeves and Stanley, 1966; Bergenholtz et al., 1982).

**FIGURE 1:**
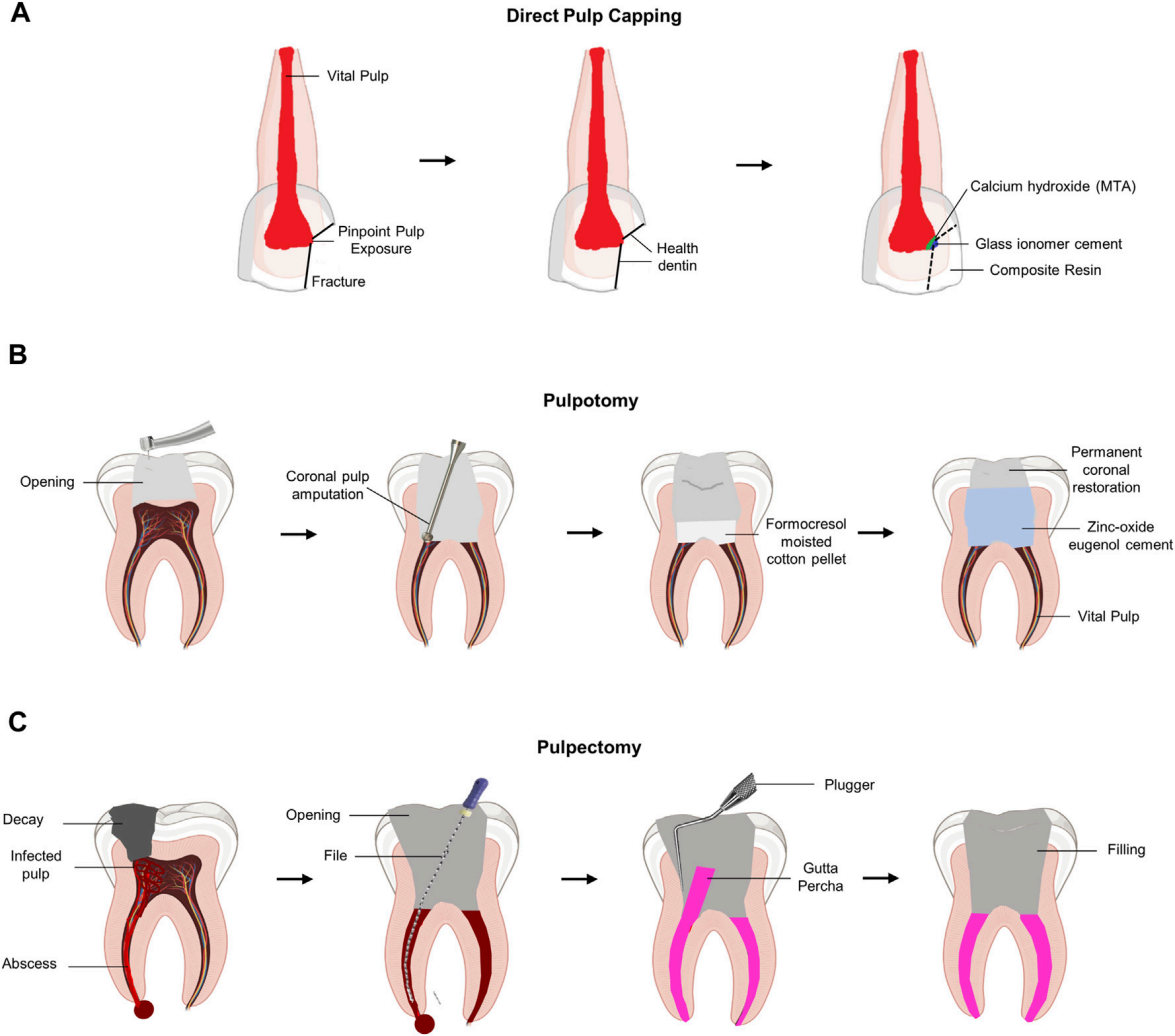
Schematic diagram of the current concept of pulp therapy. **(A)** Direct pulp capping. Pulp capping is a method used to prevent necrosis of the dental pulp when it is slightly exposed to the pin-point. Direct pulp capping covers the exposed dental pulp with a base that protects the pulp and prevents infection to maintain dental pulp vitality. **(B)** Pulpotomy. Pulpotomy is a minimally invasive method that is clinically considered when there is pulpitis in the absence of root pathology. **(C)** Pulpectomy. Pulpectomy refers to a root canal treatment that removes irreversibly infected or necrotic pulp tissue.

Direct pulp capping or pulpotomy is clinically performed when the pulp is exposed to inflammation to maintain the vitality of the pulp. Direct pulp capping with dental biomaterial was performed to protect the exposed vital pulp by promoting restorative dentin formation (Figure 1A). Therefore, the primary purpose of pulp capping is to protect the exposed pulp tissue from external stimuli, such as bacteria. Therefore, pulp capping does not involve any process to remove the pulp tissue. In contrast, pulpotomy involves the removal of an exposed area (2–3 mm) of the infected pulp tissue (European Society of Endodontology, 2006) (Figure 1B). Pulpotomy is a treatment method based on histological research findings that irreversible pulpitis causes inflammation of the coronal pulp, whereas inflammation of the pulp tissue in the root chamber is rare (Ricucci et al., 2014). Although pulp chamber pulpotomy is mainly used to allow apexogenesis in immature teeth, recent reports suggest that it may have promising long-term results in mature teeth as well (Simon et al., 2013; Taha and Khazali, 2017). However, if the bacterial infection and inflammatory reaction of the pulp continue without proper treatment, the pressure inside the pulp chamber increases significantly, resulting in ischemia of the pulp tissue with severe pain. To reduce the patient’s pain by lowering the pressure inside the pulp chamber, pulpectomy was performed to remove all pulp tissue (Figure 1C).

## Mesenchymal Stem Cells

MSCs, which have the potential for self-renewal and multiple differentiation, play an essential role in organ development and repair (Uccelli et al., 2008; Bianco et al., 2013; Frenette et al., 2013). In various studies involving animal models and clinical trials, MSCs have received significant attention in regenerative medicine because of their tremendous potential to regenerate damaged tissues, including bones and teeth (Volponi et al., 2010; Daley, 2012; Grayson et al., 2015; Trounson and McDonald, 2015). MSCs have been proposed as an attractive cell source because of their ability to differentiate into osteoblasts or odontoblasts, to cryopreserve, and to modulate systemic immunity as well as avoid ethical disputes during the harvesting process, (Volponi et al., 2010; Daley, 2012; Grayson et al., 2015; Gao et al., 2017). Moreover, MSCs grow easily *in vitro* and are thus suitable for conducting various experiments in the field of regenerative medicine. MSCs can be identified by expressing cell surface markers such as CD73, CD90, and CD105 without expressing hematopoietic cell markers such as CD11b, CD34, and CD45 (Dominici et al., 2006). Human MSCs are multipotent cells isolated from various tissues, but the most common source tissues are bone marrow and adipose tissue (Haynesworth et al., 1992; Pittenger et al., 1999; Halvorsen et al., 2000; Zuk et al., 2001).

Bone marrow-derived MSCs have been extensively studied for bone regeneration because they strongly regulate bone homeostasis by regulating osteoblast differentiation and osteoclast activity (Frenette et al., 2013; Liu et al., 2014; Fernandes and Yang, 2016; Guo et al., 2018). With regard to bone homeostasis, cytotherapy or tissue engineering techniques have demonstrated therapeutic potential for the treatment of bone marrow-derived MSCs in osteopenia and bone defects (Shang et al., 2014; Liu S. et al., 2015; Sui et al., 2016; Sui et al., 2017). The iliac crest is mainly used to collect MSCs from bone marrow, and because bone marrow is renewable, it can be freely collected without ethical issues. However, harvesting the bone marrow is not readily accessible, as it requires conscious sedation and anesthesia, which requires monitoring by an anesthesiologist.

MSCs isolated from dental-related tissues exhibit typical MSC characteristics and have been found in various dental tissues, such as extracted teeth and adherent tissues (Gronthos et al., 2000; Gronthos et al., 2002; Miura et al., 2003; Seo et al., 2004). Unlike MSCs isolated from bone marrow, MSCs isolated from dental tissues are easily accessible because they can be isolated from wisdom teeth or healthy teeth that have been extracted for orthodontic purposes. It has been reported that the frequency of extraction of the four first premolars among orthodontic patients is as high as 8.9%–13.4% (Jackson et al., 2017). In addition, based on the dentist’s value and empirical evidence, many asymptomatic third molars are extracted for prophylactic purposes prior to orthodontic treatment (Bastos Ado et al., 2016). Considering these points, MSCs isolated from dental-related tissues can be obtained more easily than MSCs isolated from bone marrow. The easily accessible dental-derived MSCs include periodontal ligament stem cells, stem cells from apical papilla, dental follicle cells, stem cells from human exfoliated deciduous teeth, and DPSCs.

### Periodontal Ligament Stem Cells

The periodontal ligament is a fibrous connective tissue that plays an important role in supporting teeth by anchoring them to the alveolar bone (Chen et al., 2012). Periodontal ligament stem cells isolated from the periodontal ligament are known to play a role in maintaining the function of periodontal tissue and regenerating the structure (Seo et al., 2004). *In vitro* experiments showed that periodontal ligament stem cells expressed cementoblast/osteoblast markers in culture and could be mineralized. In case of periodontal tissue defects, locally transplanted periodontal ligament stem cells migrate to and repair the defect, suggesting the possibility of periodontal tissue regeneration (Liu et al., 2008; Ding et al., 2010).

### Stem Cells from the Apical Papilla

The apical papilla tissue is located at the tip of the growing tooth root because it exists only during the development of the tooth root (Sonoyama et al., 2006; Huang et al., 2008; Sonoyama et al., 2008). The differentiation of stem cells from the apical papilla into odontoblasts and osteoblasts was confirmed *in vitro*, and the possibility of regeneration into cementum and periodontal ligament-like complexes was indicated *in vivo* (Han et al., 2010).

### Dental Follicle Cells

The dental follicle is a loose connective tissue derived from the ectomesenchyme that surrounds the dental papilla and the enamel of the developing tooth germ before eruption. Dental follicle cells, including progenitors of cementoblasts, periodontal ligaments, and osteoblasts, were found to differentiate into cementum *in vitro* (Kemoun et al., 2007; Yao et al., 2008) and *in vivo* in an experiment that used implants (Handa et al., 2002). In addition, dental follicle cells not only regenerate periodontal ligament-like tissues upon transplantation *in vivo* but also regenerate periodontal tissues through epithelial–mesenchymal interactions (Yokoi et al., 2007; Bai et al., 2011). Dental follicle cells are attracting attention in regenerative medicine because they maintain the characteristics of MSCs and form periodontal tissues even when sub-cultured more than other stem cells (Guo et al., 2012).

### Stem Cells from Human Exfoliated Deciduous Teeth

A cell population exhibiting the characteristics of MSCs was isolated from the pulp tissue of human exfoliated deciduous teeth and named “stem cells from human exfoliated deciduous teeth” (Miura et al., 2003). Stem cells from human exfoliated deciduous teeth can differentiate into osteoblasts *in vitro* (Su et al., 2016), and their differentiation into dentin-like tissues upon transplantation *in vivo* indicates that they have the potential for pulp regeneration (Miura et al., 2003; Shi et al., 2005; Cordeiro et al., 2008). Notably, stem cells from human exfoliated deciduous teeth showed a higher proliferation rate, osteogenic differentiation ability, and osteo-inductive potential compared to those of DPSCs and bone marrow-derived MSCs (Nakamura et al., 2009; Kunimatsu et al., 2018). Moreover, stem cells from human exfoliated deciduous teeth are considered an attractive cell source for bone and tooth regeneration because they can cryopreserve and maintain their differentiation potential even after cryopreservation (Ma et al., 2012).

### Dental Pulp Stem Cells

**FIGURE 2:**
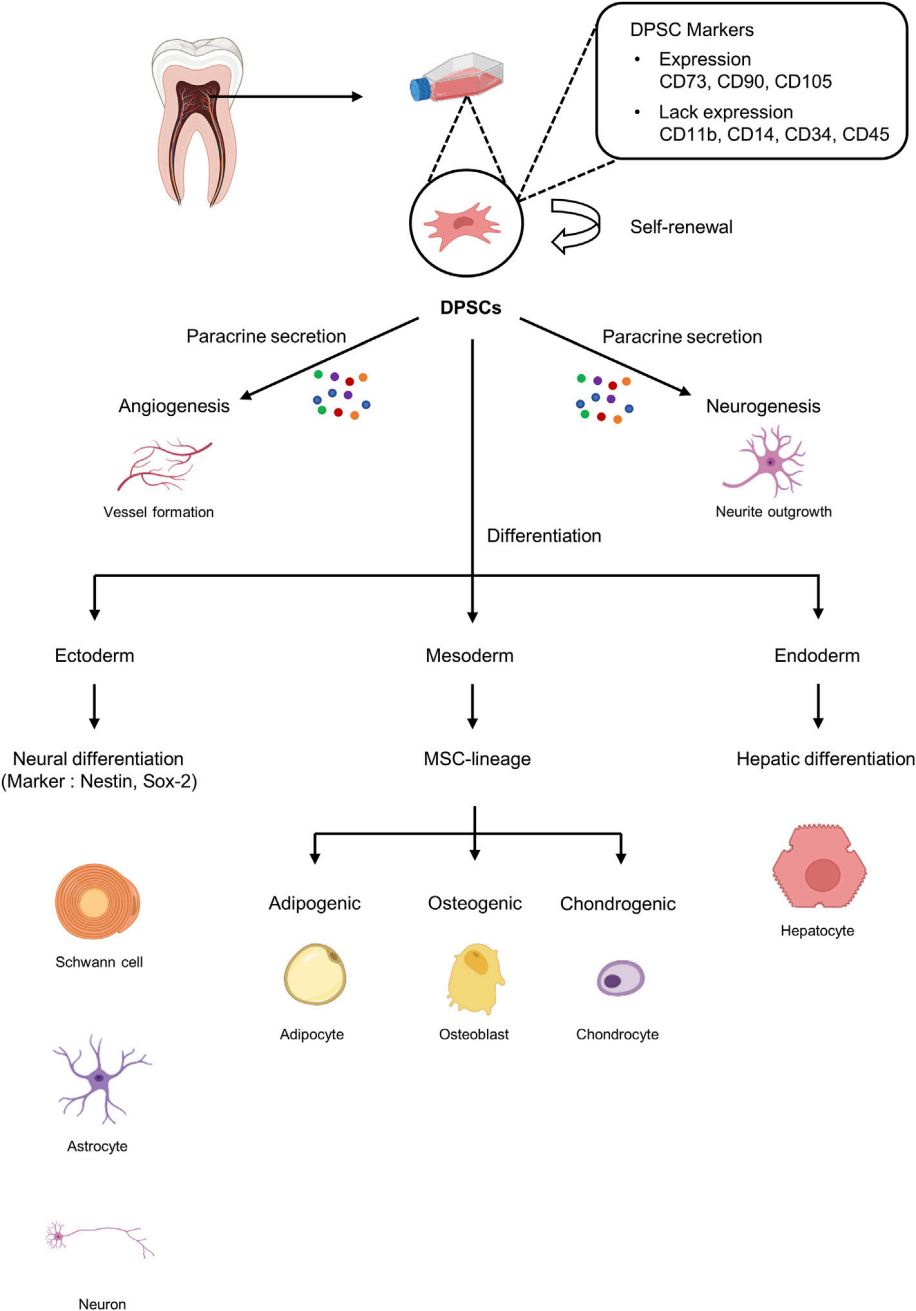
Characteristics of dental pulp stem cells (DPSCs). DPSCs can be isolated from dental pulp tissue and express markers similar to those on mesenchymal stem cells (MSCs). DPSCs can undergo self-renewal and have the potential to differentiate into ectoderm, mesoderm, and endoderm.

DPSCs are cells with MSC-like characteristics isolated from dental pulp that play a role in periodontal tissue repair and regeneration (Gronthos et al., 2000). DPSCs play an essential role in dentin repair and postnatal tooth homeostasis by differentiating into odontoblasts (Gronthos et al., 2002; Shi and Gronthos, 2003; Laino et al., 2005). Furthermore, because DPSCs are of neural origin, they can differentiate into glial cells and neurons and have also been shown to exhibit the ability to secrete neurotrophic factors that play a role in neurite outgrowth and neuroprotection (Arthur et al., 2008; Ratajczak et al., 2016) (Figure 2). Importantly, DPSCs possess a strong angiogenic capacity to generate capillary-like structures by secreting angiogenesis regulators under certain environmental conditions (Ratajczak et al., 2016) (Figure 2). Taken together, DPSCs with excellent neurodifferentiation and strong angiogenic potential, which are the most important factors for functional pulp regeneration, are the optimal cell source for dental pulp regeneration.

## Dental Pulp Stem Cells as a Source of Pulp Regeneration

The pulp tissue receives blood vessels from the apical foramen to maintain vitality and is innervated to provide sensation to the teeth. Therefore, the strong angiogenic and neurogenic potential of MSCs is essential for the successful regeneration of pulp tissue. Therefore, the regenerated pulp tissue must 1) have a cell density and structure similar to those of the original pulp, 2) generate new dentin at a controlled rate similar to that of the original pulp, 3) have blood vessels formed and connected, and 4) innervated nerves (Fawzy El-Sayed et al., 2015). DPSCs have distinctive neurovascular differentiation characteristics, suggesting that they may serve as the best candidates for pulp tissue regeneration (Gronthos et al., 2000). In addition, the pulp microenvironment maintains dynamic homeostasis, and these microenvironments must be closely mimicked during pulp regeneration. Thus, DPSCs surrounding the neurovascular bundle may be most suitable for pulp tissue regeneration (Shi and Gronthos, 2003; Zhao et al., 2014). Therefore, we focused on DPSCs and summarized their stemness, clinical application, immunomodulatory properties, and cryopreservation characteristics.

### Stemness

DPSCs are ectoderm-derived MSCs originating from the cranial neural crest cells. DPSCs have MSC-like properties, including fibroblast-like morphology and the ability to adhere to and proliferate on plastic surfaces, and exhibit MSC-like colony formation (Dominici et al., 2006; Martens et al., 2013). Similar to MSCs, DPSCs express specific markers such as CD73, CD90, CD105, and STRO-1 but not hematopoietic markers such as CD11b and CD19 (Mattei et al., 2015). However, DPSCs are a heterogeneous population that also express a variety of other markers (Figure 2).

For successful tissue engineering, forming a rapid vascular network with the host circulatory system to supply the necessary oxygen and nutrients and remove waste products is essential (Jain et al., 2005). In relation to angiogenesis, DPSCs significantly upregulate pericyte markers such as NG2, platelet-derived growth factor receptor β (PDGFRβ), and α-smooth muscle actin (α-SMA) but do not express endothelial markers such as von Willebrand factor or CD31 (Janebodin et al., 2013). It has been reported that DPSCs support angiogenic and vasculogenic processes not only by secreting pro-angiogenic factors but also by direct differentiation into pericytes and endotheliocytes. Angiogenic potential has been established through pro-angiogenic factors like vascular endothelial growth factor (VEGF), platelet-derived growth factor (PDGF), monocyte chemoattractant protein-1 (MCP-1), basic fibroblast growth factor (bFGF), and endothelin-1 (EDN1) secreted by DPSCs, and through these factors, DPSCs act as pericyte-like cells to stabilize blood vessels (Bronckaers et al., 2013; Janebodin et al., 2013).

The co-transplantation of endothelial progenitor cells and perivascular cells can form functional micro-vessels *in vivo* (Melero-Martin et al., 2007). Moreover, further administration of DPSCs can stabilize the pre-existing vasculature-like structure formed by human umbilical vein endothelial cells (HUVECs) and increase their longevity (Dissanayaka et al., 2012). When co-injected with HUVECs, DPSCs showed perivascular characteristics that contributed to angiogenesis (Nam et al., 2017). As such, it has been established that DPSCs are often closely associated with blood vessels, adopting the location and function of pericytes.

DPSCs derived from the cranial neural crest have neural properties, and in this regard, they are known to express nestin, a neural progenitor marker, and glial fibrillary acidic protein (GFAP), a glial marker (Davidson, 1994). DPSCs can differentiate into neural cells (Stevens et al., 2008) and glial cells (Gronthos et al., 2002) as well as neuronal nuclei (NeuN), neuron-specific markers that indicate neuronal differentiation capacity under neuronal induction conditions (Gronthos et al., 2002). DPSCs cultured in neuronal inductive media containing growth factors such as glial cell line-derived neurotrophic factor (GDNF) and brain-derived neurotrophic factor (BDNF), are known to differentiate into neuron-like cells (Chang et al., 2014). Moreover, it was recently reported that optogenetic stimulation not only increases the vitality of DPSCs but also promotes differentiation to neuron-like cells (Niyazi et al., 2020).

In DPSCs, like other stem cells, side population (SP) cells characterized by a low level of Hoechst33342, a DNA-binding fluorescent dye, were found. Among the SP populations, the CD31^−^/CD146^−^ population is considered a promising population owing to the high expression of neurotrophic factors such as BDNF and nerve growth factor (NGF) and angiotrophic factors such as VEGF-A (Nakashima et al., 2009).

Therefore, DPSCs have cell characteristics suitable for angiogenesis and neurogenesis, which are essential for pulp regeneration.

### Immunomodulation Properties

**FIGURE 3:**
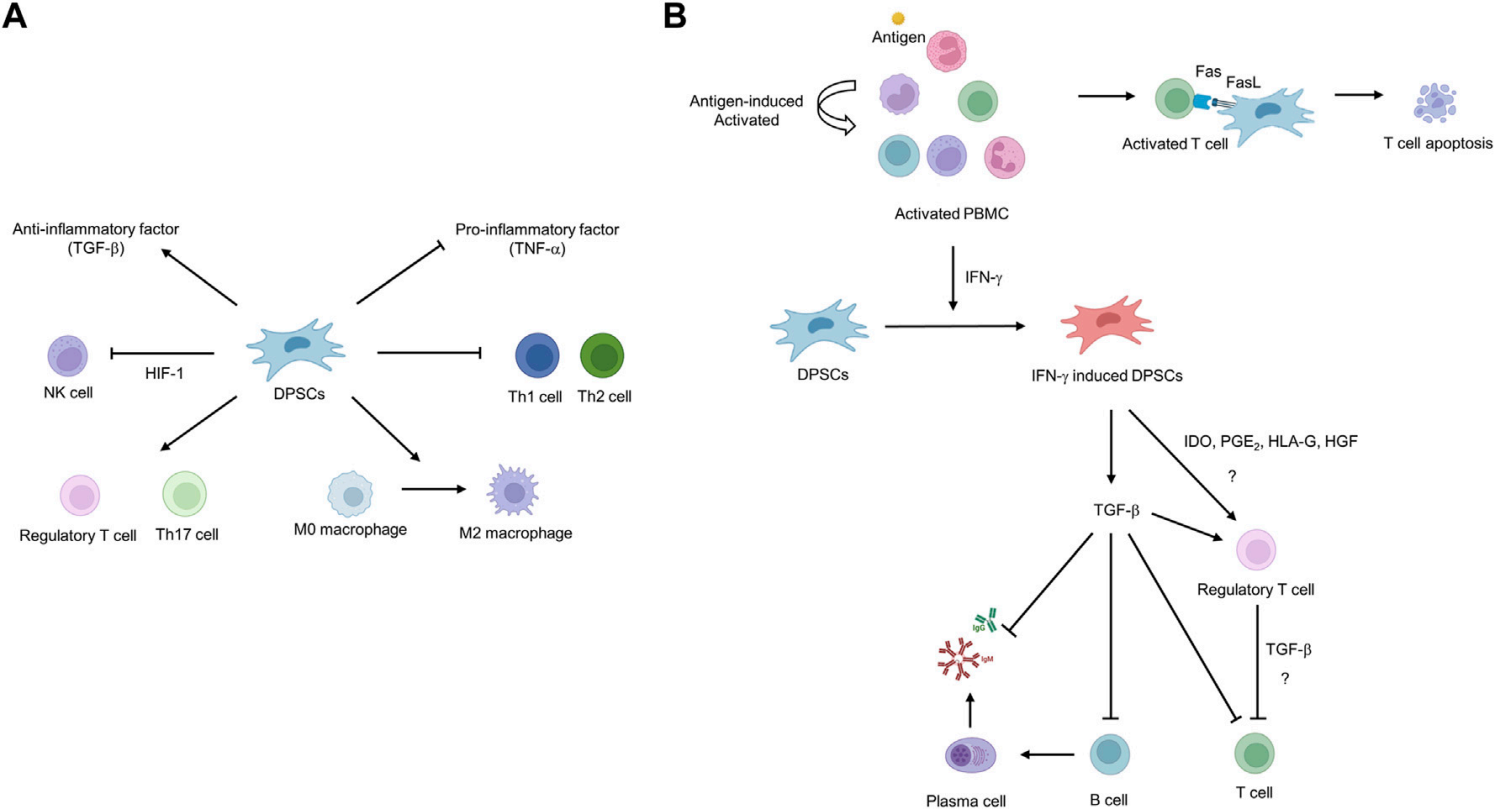
Schematic diagram of the immunosuppressive potential of dental pulp stem cells (DPSCs). **(A)** DPSCs can inhibit the proliferation of natural killer (NK), T helper 1 (Th1), and T helper 2 (Th2) cells and the secretion of pro-inflammatory factors such as TNF-α. In addition, DPSCs induce the proliferation of regulatory T cells (Tregs)/T helper 17 (Th17) cells and differentiation toward macrophage M2 phenotype and secrete anti-inflammatory factors such as TGF-β. **(B)** Fas ligand expressed in DPSCs induces T-cell apoptosis through Fas apoptotic pathway. IFN-γ secreted from hyper-activated T cells primes DPSCs to secrete TGF-β, resulting in immunosuppressive ability.

Autologous DPSCs are considered a suitable source of cells for cell-based regenerative medicine but have limitations in that the uninfected teeth must be extracted and cryopreserved in a timely manner and used within the cryopreservation period. In contrast, using allogeneic cells has the advantage that when clinically applicable DPSCs are secured, a cell bank can be created and appropriately applied to patients in need at any time. However, allogeneic cell use can induce immune rejection by the host immune system due to a major histocompatibility complex (MHC) mismatch. DPSCs have effective and potent immunomodulatory functions to address immune rejection, suggesting their potential for regenerative medicine using allogeneic cells. Studies have primarily demonstrated the immunosuppressive properties of DPSCs through *in vitro* cell co-culture. Co-culture of stimulated T cells with DPSCs inhibits T-cell proliferation through the formation of regulatory T cells (Tregs), suggesting that Tregs may play a pivotal role in the immunosuppressive properties of DPSCs (Pierdomenico et al., 2005; Demircan et al., 2011; Hong et al., 2017) (Figure 3). Another study reported that DPSCs suppressed Th1 and Th2 subsets of CD4^+^ T cells while increasing the proliferation of Treg and Th17 subsets (Ozdemir et al., 2016) (Figure 3A). In contrast, our group reported that Tregs are not directly related to the immunosuppressive properties of DPSCs. We reported that T cells are activated to secrete IFN-γ, which primes DPSCs to release TFG-β, thereby exhibiting immunosuppressive activity (Kwack et al., 2017) (Figure 3B). There was also a report that the Fas ligand expressed in DPSCs induces T-cell apoptosis through the Fas apoptotic pathway, resulting in the immunosuppressive property of DPSCs (Zhao et al., 2012). DPSCs participate in immune responses by interacting with macrophages and natural killer (NK) cells in addition to T cells. Transplanting DPSCs into unilateral hindlimb skeletal muscle showed that DPSCs could interact with macrophages to promote polarization toward the anti-inflammatory M2 phenotype (Omi et al., 2016). NK cells are considered important mediators in cell therapy because they efficiently lyse transplanted autologous and allogeneic MSCs (Spaggiari et al., 2006). However, DPSCs have been shown to increase resistance to NK cell lysis by overexpressing hypoxia-inducible factor 1 alpha (HIF-1α), thereby increasing the potential *in vivo* lifespan of transplanted DPSCs (Martinez et al., 2017).

DPSCs are known to modulate inflammatory factors, which downregulate the production of pro-inflammatory factors such as TNF-α, while upregulating the secretion of anti-inflammatory factors such as TGF-β (Demircan et al., 2011). Among them, TGF-β has been reported to be expressed in DPSCs as an immunosuppressive regulator and anti-inflammatory factor. It has been reported that DPSCs promote nerve repair and regeneration by releasing TGF-β in response to nerve damage and suppressing the acute immune response (Luo et al., 2018). These findings suggest that DPSCs may be a suitable source for allogeneic transplantation, as they not only exhibit immunomodulatory properties by regulating immune cell proliferation and cytokine production but are also involved in regulating inflammation.

However, activating immune cells *in vitro* is an artificial process and has a limitation in that it can hardly represent complex immune responses that are actually generated *in vivo*. The immunomodulatory properties of DPSCs are strongly influenced by the surrounding microenvironment and are generally not observed in steady-state quiescent DPSCs. MSCs are primed by inflammatory cytokines such as TNF-α and IFN-γ, which are released by activated immune cells. Primed MSCs, in turn, greatly enhance their immunosuppressive potential. Likewise, our group emphasized the importance of the surrounding microenvironment by demonstrating that the immunosuppressive effect disappeared when DPSCs were incubated with IFN-γ antibody to neutralize IFN-γ (Kwack et al., 2017). Further research is needed to understand the exact mechanism of the immunosuppression of DPSCs that occurs *in vivo*. However, according to a recent preclinical study of allogeneic transplantation of DPSCs after pulpectomy of canine incisors, the absence of side effects following transplantation of allogeneic mismatched DPSCs suggests that it exhibits immunosuppressive ability even *in vivo* (Iohara et al., 2018). Moreover, a recent report that the immunomodulatory effect of undifferentiated DPSCs is maintained during osteogenic differentiation supports the strong immunomodulatory ability of DPSCs (Hossein-Khannazer et al., 2019).

### Cryopreservation

Although DPSCs have regenerative activity for clinical applications, it is known that DPSCs isolated from the teeth of elderly patients or patients with systemic diseases such as systemic lupus erythematosus, rheumatoid arthritis, or diabetes have reduced bioactivity (Zhang J. et al., 2015). In particular, in elderly patients, not only does the pulp tissue shrink due to physiological secondary dentinogenesis and pathological tertiary dentinogenesis but also mineralization, such as pulpal stone, limits the acquisition of DPSCs, thus limiting its use. In addition, it is almost impossible to obtain and use the dental pulp of uninfected young patients on an as-needed basis. Therefore, cell banking is an essential technology for storing DPSCs in clinically appropriate conditions with minimal cell or tissue damage and applying them through immediate cell expansion when clinically needed (Woods et al., 2009). Cryopreservation is the process of maintaining cell viability by freezing and storing them at extremely low temperatures where biochemical reactions do not occur (Mullen and Critser, 2007). However, since cells are easily exposed to stressful conditions during cryopreservation, which leads to cryoinjury, research on how to prevent damage has been in progress for a long time. Cryoinjury can occur either through direct mechanical action due to the formation of ice crystals or by secondary effects due to changes in osmotic homeostasis (Pegg, 2015). To prevent such damage, cryoprotectants are used, of which the most widely used is dimethyl sulfoxide (DMSO), which penetrates through the cell cytoplasmic membrane and prevents the formation of ice crystal nuclei. However, because DMSO itself adversely affects cells (causes cytotoxic effects), it is necessary to limit the concentration used for cell preservation. In general, it is recognized that a concentration of 10% or less is slightly toxic, and studies on the concentration of DMSO suitable for preservation of DPSCs are being conducted.

Importantly, for the application of DPSCs in regenerative medicine, cryopreservation should not affect their stemness features and multipotency. Several studies have shown that DPSCs can be cryopreserved while retaining their stem cell properties (Zhang et al., 2006; Gioventu et al., 2012). A recent study showed that DPSCs did not impair viability, proliferation, stemness, or differentiation capacity after cryopreservation at −80°C for 1 year (Pilbauerova et al., 2021). Despite these advantages, DPSCs can be cryopreserved only when an appropriate number is obtained by isolating and culturing DPSCs from pulp tissue after tooth extraction. This method takes a long time for cryopreservation, resulting in excessive labor and other costs, and there is even a risk of potential contamination by microorganisms. Therefore, there are studies moving from cell-level cryopreservation to tissue-level cryopreservation. There was no significant change in cell proliferation rates, cell growth morphology, and stem cell characteristics when pulp tissue was stored in liquid nitrogen for more than 1 year and then cultured (Han et al., 2017). Another study reported that the time lapse for cellular outgrowth was significantly reduced when 5% DMSO was used for cryopreservation of pulp tissue compared to when 10% DMSO was used without affecting other conditions (Yan et al., 2020). Therefore, it may be better to choose tissue cryopreservation over cell cryopreservation in that it positively affects the vitality of cells by reducing the time until cryopreservation, reducing the possibility of contamination, and reducing the direct toxic effects of DMSO on cells. Pulp tissue has been used as a scaffold for dental pulp regeneration (Hu et al., 2017; Song et al., 2017; Matoug-Elwerfelli et al., 2020; Bakhtiar et al., 2021), and cryopreservation as a tissue can be a good method for preserving the scaffold. In addition to these characteristics, DPSCs themselves have immunosuppressive properties (Kwack et al., 2017), and long-term cryopreservation weakens their immunogenicity (Yokomise et al., 1996), suggesting the possibility of allogeneic pulp tissue transplantation.

### Therapeutic Potential of Dental Pulp Stem Cells Related to Neurovascular Properties

Owing to the angiogenic and neurogenic potential of DPSCs, they are being studied for the treatment of various systemic diseases. In a study showing the neurodifferentiation properties of DPSCs, ectopic implantation of pulp tissue into the anterior chamber of rats resulted in innervation and upregulation of catecholaminergic nerve fiber density in the iris (Nosrat et al., 2001). In the same study, implantation of pulp tissue into hemisected spinal cords showed an increase in the number of surviving motor neurons, indicating that this effect is orchestrated by dental pulp-derived neurotrophic factors that functioned by rescuing motoneurons. Dental pulp-derived neurotrophic factors have been reported to have neuroprotective effects in Parkinson’s disease by protecting dopaminergic neurons from MPP+ or rotenone toxicity *in vitro* (Nesti et al., 2011; Gnanasegaran et al., 2017). Moreover, DPSCs have been shown to have neurotrophic effects in Alzheimer’s disease and Parkinson’s disease (Apel et al., 2009; Martens et al., 2013; Zhang X.-M. et al., 2021). In particular, human dental pulp cells express a neuronal phenotype and produce neurotrophic factors such as NGF, GDNF, BDNF, and bone morphogenetic protein (BMP)-2, suggesting that they may be potential candidates for cell-based therapy.

The angiogenic potential of DPSCs has also been studied in several disease models. Functional revascularization was induced by transplantation of the CD31^−^ CD146^−^ SP of DPSCs isolated from porcine pulp tissue into the mouse hindlimb ischemia site (Iohara et al., 2008). The SP of DPSCs not only induces angiogenesis but also promotes the migration and differentiation of endogenous neural progenitors, thereby improving ischemic brain injury after middle cerebral artery occlusion (Sugiyama et al., 2011). In addition, the angiogenic potential of DPSCs can be determined from the study results that human DPSCs induce angiogenesis and alleviate infarction in rats with acute myocardial infarction (Gandia et al., 2008). The angiogenic potential of DPSCs was also shown in a model of muscular dystrophy, where DPSCs were engrafted into the host muscle, resulting in histological improvement by enhancing angiogenesis (Pisciotta et al., 2015). In dystrophic mouse models, human dental pulp pluripotent-like stem cells were engrafted into skeletal muscle and showed integration in muscle fibers and blood vessels by secreting several growth factors involved in angiogenesis (Martinez-Sarra et al., 2017).

## The Classical Concept of Pulp Tissue Engineering

Traditionally, infected dental pulp undergoes RCT in which all dental pulp is removed, and the pulp space is filled with artificial inorganic materials. However, teeth treated with the RCT method lose their vitality and become brittle, making them susceptible to postoperative fracture. Therefore, maintaining the vitality of dental pulp is an appropriate treatment to solve these problems. With the development of tissue engineering technology and regenerative medicine, efforts are being made to regenerate pulp tissue to maintain the vitality of teeth.

The three classical elements traditionally required in regenerative medicine are stem cells, scaffolds, and signaling molecules (growth factors), and the same concept has been used for dental pulp regeneration. Briefly, the concept is to isolate and culture stem cells *in vitro*, load them onto scaffolds, and apply them *in vivo* with signaling molecules that can help stem cells to properly differentiate.

The scaffold primarily serves as a tool to support stem cells, but it can also play a role in attracting cells or promoting differentiation into specific cells by additionally loading growth factors or drugs (Brittberg et al., 1994). From a classical point of view, scaffolds are important for structural support, allow them to interact with their surrounding microenvironment, and can influence the signal pathway required for regeneration. To achieve this classical purpose, the scaffold must mechanically maintain its integrity, thereby supporting the adhesion and differentiation of stem cells to the implantation site. Additionally, the scaffold must mimic the original extracellular matrix of the tissue from which it is generated (Goldberg and Smith, 2004; Du and Moradian-Oldak, 2006).

Various exogenous growth factors have been demonstrated to enhance the migration, proliferation, and differentiation of DPSCs *in vitro*. Factors with excellent potential to induce the migration of DPSCs include bFGF (Suzuki et al., 2011; Takeuchi et al., 2015), stromal-derived factor-1 (SDF-1) (Suzuki et al., 2011; Yang et al., 2015) and granulocyte-colony stimulating factor (G-CSF) (Takeuchi et al., 2015). In addition, wnt3a (Hunter et al., 2015), G-CSF, and bFGF have been reported to promote the proliferation of DPSCs. Several factors are known to induce differentiation of DPSCs, and in particular, BMP-2 has been reported to induce their differentiation into odontoblasts (Oshima and Tsuji, 2014). Moreover, although BMP-7 had no significant effect on the recruitment of DPSCs, it induced mineralization of DPSCs (Suzuki et al., 2011), and TGF-β stimulated mineralization by differentiating DPSCs into odontoblast-like cells (Oshima and Tsuji, 2014). G-CSF was also a factor inducing dentinogenesis in DPSCs (Takeuchi et al., 2015); importantly, G-CSF is known to induce the migration, differentiation, and mineralization of DPSCs, as well as neurogenesis and angiogenesis, suggesting that it is an essential factor for pulp regeneration (Takeuchi et al., 2015). However, since the multiple actions of one factor may interfere with the sophisticated differentiation regulation for dental pulp regeneration, detailed mechanistic studies should be conducted.

## Current Approaches for Pulp Tissue Regeneration

**FIGURE 4:**
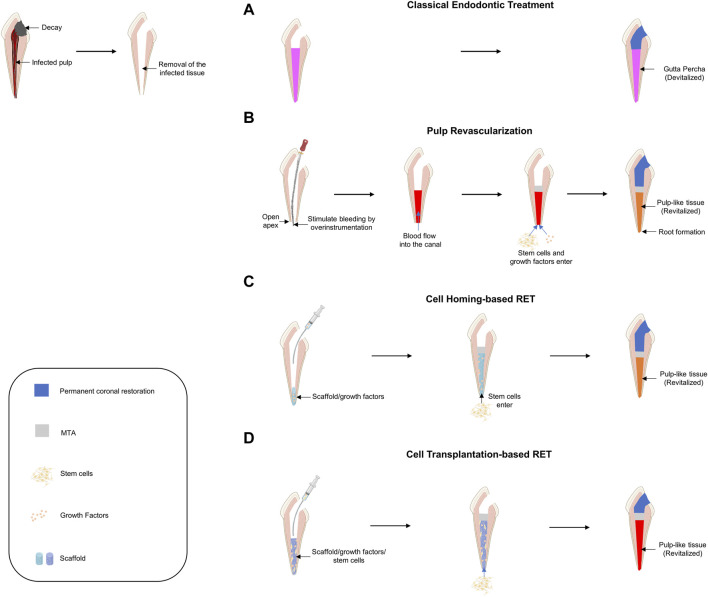
Schematic diagram of current approaches for pulp tissue regeneration. **(A)** Classical endodontic treatment. **(B)** Pulp revascularization. **(C)** Cell-homing-based regenerative endodontic treatment (RET). **(D)** Cell-transplantation-based RET.

If decayed teeth progress without proper treatment, pulp necrosis and abscesses accompanied by inflammatory pulp reactions occur. To save the infected tooth, an RCT was performed in which the entire infected pulp tissue was removed and disinfected, and the empty space was filled with artificial material (Figure 4A).

### Pulp Revascularization

Pulp revascularization is a procedure that regenerates the infected pulp tissue into pulp-like tissue by filling the root canal space with a blood clot after disinfection (Figure 4B). Because this procedure must induce a blood clot from the apical foramen, it can be used on immature teeth where the apical foramen has not yet closed. Attempts to regenerate pulp tissue by inducing blood clots into the root canal were first made in the 1960s (Ostby, 1961). If revascularization is successfully performed in immature teeth, root development of immature teeth can be completed; therefore, it has been actively studied in the field of traumatology (Skoglund et al., 1978; Galler, 2016). Many studies have been conducted, including the first case report on regenerative root canal treatment, and this treatment was adopted by the American Dental Association in 2011 and is now widely used in clinical practice (Iwaya et al., 2001; Banchs and Trope, 2004). Although pulp revascularization is similar to conventional root canal treatment in that it removes infectious agents, there are some differences in the basic concept. In RCT (or pulpectomy), to prevent re-infection, an aggressive disinfection process is performed, and artificial materials are filled in. In contrast, in pulp revascularization, mechanical debridement using an endodontic file is contraindicated to prevent damage to the root canal wall and induce the influx of stem cells located on the apical side to maintain the vitality of the tooth (Cvek, 1992; Iwaya et al., 2001). For this reason, in pulp revascularization, the use of intracanal medicaments along with the application of sufficient chemical disinfection is recommended instead of mechanical debridement. However, even if chemical disinfection is performed to protect the root canal wall, it is necessary to consider the balance between disinfection and cytotoxicity in stem cells. Thorough disinfection is important to prevent re-infection; however, an appropriate microenvironment for stem cell adhesion and differentiation is also needed for stem cell regeneration. Sodium hypochlorite, a representative chemical disinfectant, is known to be cytotoxic at a concentration of 3% or more and interferes with stem cell adhesion (Chang et al., 2001; Ring et al., 2008; Martin et al., 2014). Accordingly, the American Association of Endodontists recommends the use of a low concentration of sodium hypochlorite for pulp revascularization. Pulp revascularization faithfully follows the classical concept of tissue engineering. By inducing bleeding, MSCs from the apical side are delivered into the root canal (Lovelace et al., 2011), and the blood clot acts as a scaffold as well as a signaling molecule because of the presence of many growth factors (Shah et al., 2008; Nosrat et al., 2012). Reportedly, ethylenediaminetetraacetic acid (EDTA) can promote the differentiation of DPSCs into odontoblast-like cells by releasing various growth factors entrapped in dentin (Galler et al., 2011). Therefore, EDTA is recommended as the final irrigant. However, pulp revascularization has several limitations. As mentioned earlier, this method can only be used for immature teeth. Moreover, histological studies have shown that most tissues formed through pulp revascularization are not original pulp-like tissues but contain tissues such as periodontal, cementum, and bone-like tissues (Becerra et al., 2014). Therefore, further studies are needed to promote the formation of pulp-like tissue and to apply this method to mature teeth.

### Cell-Homing-Based Regenerative Endodontic Treatment

The basic concept of cell homing for dental regeneration is to achieve tissue regeneration through chemotaxis of host endogenous cells to damaged pulp tissue via signaling molecules, just as our body does for damaged tissue repair (Kim et al., 2010) (Figure 4C). Pulp revascularization and cell-homing-based RET is considered cell-free RET, as it is performed without exogenous cell transplantation. In pulp revascularization, blood acts not only as a scaffold but also as a source for signaling molecules, but cell homing can be applied to a scaffold that is advantageous for stem cell migration and proliferation and can load desired signaling molecules together. Because the cell-homing strategy is to create a suitable environment for the induction, differentiation, and proliferation of endogenous stem cells capable of regenerating pulp-dentin, it is important to identify endogenous cell sources from a therapeutic point of view. Because stem cells (DPSCs) are present in the dental pulp, the source of the cells depends on whether the vital pulp is preserved in the root canal. Pulpotomy is a dental procedure that removes the pulp of a tooth in the crown and leaves the pulp in the root canal as intact vital pulp. It has been mainly used for the normal development of the root by preserving pulp in immature teeth; however, it can also be performed on mature teeth. If the cell-homing strategy is used after pulpotomy, the DPSCs that exist in the immediate vicinity are mainly homing and can regenerate pulp dentin, an intrinsic ability under the influence of signaling molecules (Shi et al., 2020). However, it is known that in pulpectomy, which extirpates the entire pulp tissue, stem cells from apical papilla, and periodontal ligament stem cells, which are mainly located in the apical foramen, are homed (Liu J.-Y. et al., 2015; He et al., 2017).

The pulp tissue regeneration experiment using the cell-homing-based technique is mainly performed by transplanting human teeth into animal models. Briefly, after RCT of human extracted teeth, different types of signaling molecules are combined on various scaffolds and applied to empty root canals. The grafts are transplanted into animal models to evaluate pulp regeneration. Early studies mainly focused on signaling molecules. Pulp-like tissue has been reported to be regenerated in a mouse model using a combination of bFGF, PDGF (factors for cellular chemotaxis), NGF (for neural growth), VEGF (for angiogenesis), and BMP7 (for odontoblast differentiation and mineralization) (Kim et al., 2010). Although the study by Kim et al. (2010) used an ectopic model, it is significant as a starting point for demonstrating a clinically accessible cell-homing approach for pulp regeneration in humans. Experiments were then conducted using a single molecule to determine which signaling molecule is most essential for pulp regeneration. Early research showed that pulp-like tissue was regenerated by injecting bFGF alone, suggesting that the signaling molecule that triggers stem cell recruitment plays the most important role (Suzuki et al., 2011). Another study found that there was no significant difference in the pulp regeneration effect between bFGF and G-CSF application and reported that bFGF could be replaced with G-CSF (Takeuchi et al., 2015). As a result of these studies, research was conducted mainly on signaling molecules that can home stem cells to the empty pulp space, and factors such as SDF-1 (Yang et al., 2015; Zhang L. X. et al., 2015) and stem cell factor (SCF) (Ruangsawasdi et al., 2017) have also been reported to be effective.

Cell-homing strategies have the advantage of not requiring isolation or manipulation of stem cells *in vitro*, making them more economical and may be easier to perform clinically. However, the cell-homing strategy also has some limitations. If the apical papilla or follicle is damaged due to severe inflammation accompanied by pulpal necrosis, it may be difficult to sustain the root development of immature teeth because there are not enough stem cells to support odontoblast differentiation or dentin formation. Likewise, even when cell-homing-based RET is performed on mature teeth, treatment may not be successful if there are not enough stem cells, and the outcome of treatment cannot be predicted because it is impossible to determine the status of stem cells in the apical papilla or dental follicle. Therefore, numerous aspects still need to be addressed to obtain applicable and predictable results in clinical practice. Despite the great advances in dental pulp regeneration through cell-homing-based RET over the past few years, further investigation and development are needed.

### Cell-Transplantation-Based Regenerative Endodontic Treatment

The basic concept of cell-transplantation-based RET is the transplantation of exogenous stem cells onto scaffolds with signaling molecules for tissue regeneration (Demarco et al., 2011) (Figure 4D). For RET, a cell transplantation strategy based on the classical concept of tissue engineering was first proposed for pulp tissue regeneration and has made remarkable progress. Based on the classical concept of tissue engineering, early experiments and continuous animal studies have been conducted to investigate the effect of stem cell implantation on pulp regeneration (Dissanayaka et al., 2015; Abbass et al., 2020; Ahmed et al., 2020). In a preliminary study in which autologous DPSCs were transplanted together with Gelfoam as a scaffold for immature permanent incisors of canines, it was found that pulp-like tissues, including dentin-like tissues and blood vessels, were regenerated (Wang et al., 2013). Another study reported that autologous DPSCs with platelet-rich fibrin promoted the regeneration of pulp-dentin like tissue in dogs (Chen et al., 2015). Further animal studies have shown that human DPSCs, along with platelet-derived growth factor, have successfully induced pulp-like tissue by applying them to the empty pulp space of rats (Cai et al., 2016). A study in which a chitosan hydrogel scaffold containing autologous DPSCs and growth factors was applied to immature necrotic permanent teeth with apical periodontitis in dogs confirmed that root maturation was complete histologically and radiologically, as well as regeneration of pulp and dentin-like tissues (El Ashiry et al., 2018).

In cell-transplantation-based RET, various scaffolds have been used to allow the applied stem cells to promote attachment, proliferation, and differentiation. Gelfoam was used as a scaffold for applying autologous DPSCs in dogs (Wang et al., 2013), and an injectable nanopeptide hydrogel was also used to apply porcine DPSCs (Mangione et al., 2017). In addition, it was reported that the gelatin-based scaffold was histologically and radiologically more effective than the fibrin-based scaffold when human DPSCs were placed on gelatin- or fibrin-based scaffolds and transplanted into minipigs (Jang et al., 2020). Furthermore, to use the tissue most similar to the original tissue as a scaffold, decellularized swine dental pulp tissue was used as a scaffold and applied to human DPSCs, and the regeneration of pulp-like tissue was confirmed histologically (Hu et al., 2017). In addition, scaffold-free RET, which can replace the role of scaffolds by constructing cells in three dimensions, is being studied. Pulp-like tissue regeneration was achieved by transforming canine DPSCs into cell sheet fragments and applying them along with the signaling molecule platelet-rich fibrin (Chen et al., 2015). In addition, it was confirmed that the pulp-like tissue was regenerated by subcutaneously implanting human DPSCs into three-dimensional (3D) cell constructs without signaling molecules or scaffolds in immunodeficient mice (Itoh et al., 2018). A recent study revealed that 3D cell sheets could enhance the therapeutic potency of MSCs, suggesting the possibility that cell sheets could replace scaffolds (Bou-Ghannam et al., 2021).

In cell-transplantation-based RET, autologous stem cells have been used in many animal studies, and all clinical trials performed using this method yielded successful results (Kim, 2021). Therefore, the cell-transplantation-based RET method appears to be the most appropriate for clinical applications. In addition, because stem cells with suitable characteristics are directly applied together to induce pulp-like tissue, the limitation of stem cell induction, as in cell-homing-based RET, can be overcome. However, a major obstacle to autologous stem cell transplantation is the availability of pulp tissue. The ethically best available methods for isolating DPSCs required for pulp regeneration are wisdom teeth or orthodontic extraction teeth. It is difficult to extract wisdom teeth or perform orthodontic treatment to treat irreversible pulpitis. In addition, the scaffolds and signaling molecules used in each study were slightly different; therefore, more research on the best combination is needed. Collectively, cell-transplantation-based RET in dentistry for dentin pulp tissue regeneration still faces challenges. Future strategies should be directed toward creating a suitable regenerative microenvironment using an ideal combination of signaling molecules and scaffolds that are most suitable for pulp tissue regeneration (Mangione et al., 2017).

## Allogeneic Transplantation

Stem cell therapy has been proposed as an effective regenerative technology to restore the function of teeth that have lost function due to pulpitis. Several specific regeneration methods have been introduced, and autologous transplantation of a subpopulation of DPSCs (pulp-derived CD31^−^ SP cells and pulp-derived CD105^+^ cells treated with SDF-1) have been successful in demonstrating the possibility of pulp regeneration (Iohara et al., 2011; Ishizaka et al., 2012). Subsequently, transplantation of DPSCs with autologous platelet-rich fibrin successfully regenerated pulp-like tissue and induced the deposition of regenerated dentin (Chen et al., 2015). In a preclinical study, the safety and efficacy of autologous DPSC transplantation therapy were demonstrated by harvesting and culturing DPSCs under good manufacturing practice conditions and applying them along with G-CSF (Iohara et al., 2013). Moreover, a recent human clinical study suggested that autologous DPSC transplantation is safe and may effectively induce pulp regeneration (Nakashima et al., 2017). Although autologous DPSC transplantation has demonstrated some efficacy and potential for tissue regeneration, certain limitations still need to be overcome, the biggest limitation being the presence of unnecessary teeth, such as wisdom teeth, to regenerate the pulp of a specific tooth with autologous DPSC transplantation. This limitation is particularly noticeable in elderly patients since there is a high probability that elderly patients do not already possess unneeded teeth. Even if elderly patients have usable teeth for transplantation, their DPSCs may show dysfunction due to aging. A recent study reported that DPSCs exhibit typical senescence features, such as reduced proliferation, reduced differentiation potential, and enlarged cell shape with aging (Yi et al., 2017). In addition, osteogenic potential decreases in aged human DPSCs (Yi et al., 2017; Iezzi et al., 2019), and the expression of dentin matrix acidic phosphoprotein 1 and dentin sialophosphoprotein, key markers of odontogenic differentiation, decreases with age (Iezzi et al., 2019). Various studies have proven that neurogenic potential, which is one of the essential factors for pulp regeneration, also decreases with age (Martens et al., 2012; Feng et al., 2013). However, it is not efficient for individual patients to bank DPSCs at an early age and store them until needed. Moreover, the storage, safety, and quality control costs are high. Therefore, allogeneic DPSC transplantation, which is stored whenever unwanted teeth are found in young patients and applied to patients in need, saves time and cost, and is beneficial for quality control (Collart-Dutilleul et al., 2015). Therefore, cell banks are essential to overcome the limitations of autologous transplantation and to successfully perform allogeneic transplantation. Many studies have shown that DPSCs can be cryopreserved without significant cell damage (Zhang et al., 2006; Gioventu et al., 2012). In addition, tissue cryopreservation, which is economically advantageous because it reduces the time and money required to isolate and incubate cells, has been shown to have no effect on cells (Han et al., 2017). Moreover, tissue cryopreservation for more than 1 year is likely to significantly reduce the cost of autologous cell-transplantation-based RET. Notably, systematizing the collection, banking, and application of pulp tissue are expected to reduce costs and simplify clinical application procedures.

A concern with the use of allogeneic cells is that immune rejection may occur in the host due to a MHC mismatch. The reason that allogeneic MSCs can be applied despite these concerns is that MSCs themselves have low immunogenicity and immunosuppressive properties. The low immunogenicity of MSCs is attributed to the low expression of class I MHC (MHC-I) proteins and costimulatory molecules and lack of expression of MHC-II proteins (Pittenger et al., 1999; Le Blanc et al., 2003). Therefore, MSCs do not exhibit cytotoxic effects on host immune cells (Jones and McTaggart, 2008) and have the advantage of being able to perform transplantation without considering MHC (Ankrum et al., 2014). MSCs and DPSCs exhibit low immunogenicity and can induce immune tolerance in the host (Iohara et al., 2018). Moreover, although the mechanism is still controversial, the immunomodulatory properties of DPSCs increase their potential as a source for allogeneic transplantation. This immunosuppressive property suggests that even if the host’s immune response occurs during allogeneic transplantation by other factors, it can be overcome, and transplantation can be successful. Based on these characteristics of DPSCs, it was recently reported that the transplantation of allogeneic DPSCs in dogs was successfully performed (Iohara et al., 2018). In this study, allogeneic transplantation DPSCs mismatched with dog leukocyte antigen (DLA) did not show toxicity and showed similar effects to DLA-matched DPSCs in pulp tissue regeneration. Thus, allogeneic “off-the-self” therapies can achieve the goal of clinical stem cell-based therapy to maintain long-term stability by inducing universal cell donor adoption, banking donated cells, and timely delivery of appropriate cells for patients in need (Telukuntla et al., 2013; Heathman et al., 2015).

## Conclusion

**FIGURE 5:**
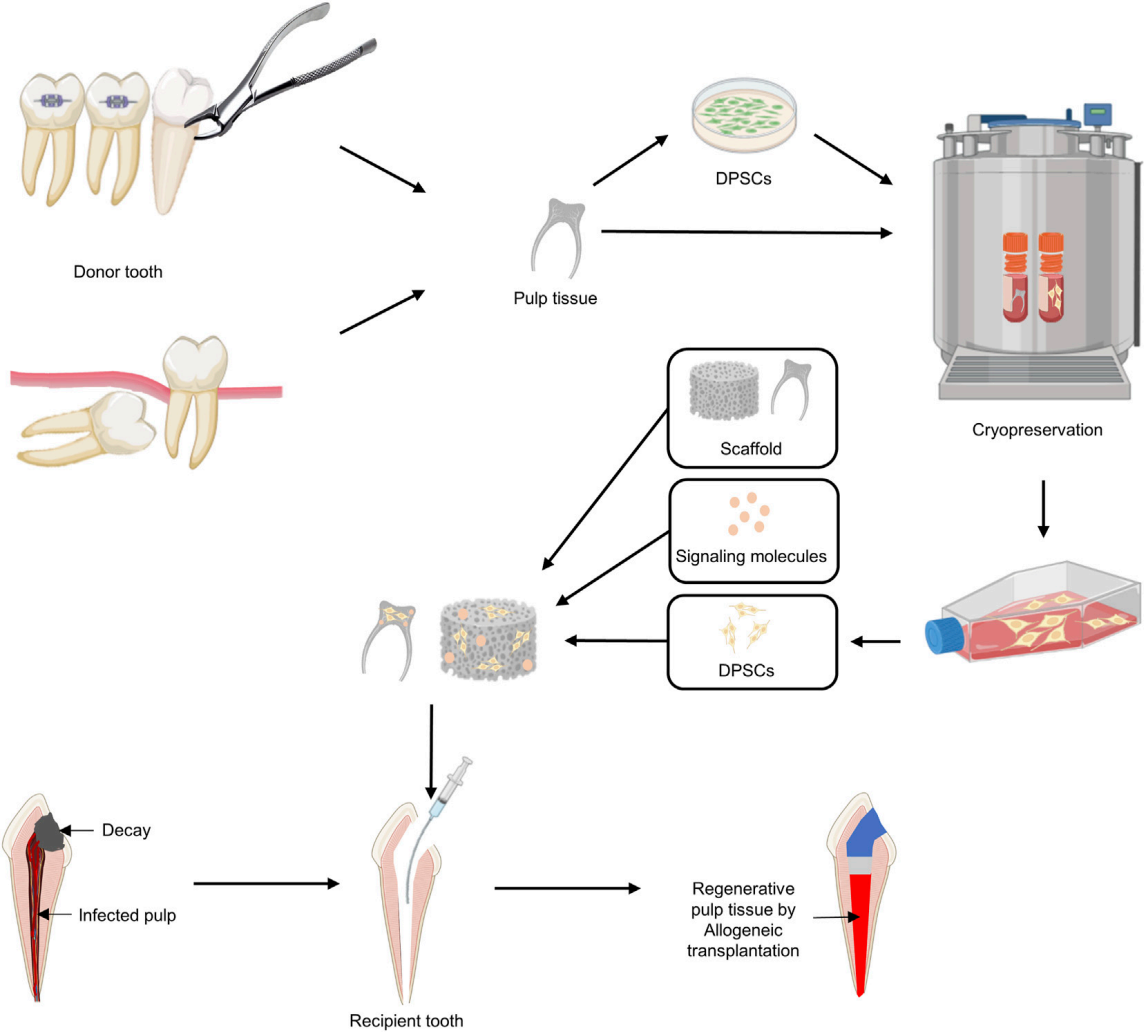
A proposed strategy using allogeneic DPSC transplantation for pulp regeneration. 1) Dental pulp tissue is removed from an uninfected orthodontic extraction tooth or wisdom tooth (donor). 2) After isolating and culturing DPSCs from dental pulp, cryopreservation of DPSCs or dental pulp itself is performed. 3) DPSCs are expanded using cryopreserved DPSCs or dental pulp tissues according to the patient’s treatment plan for pulpectomy. 4) Signaling molecules and DPSCs are added to the appropriate scaffold and applied to the disinfected pulp space.

One of the challenges facing modern dentistry is not only to remove the infected pulp but also to maintain the pulp so that it can regain vitality (Miran et al., 2016; Yang et al., 2016). The goal of pulp regenerative treatment for infected pulp is to restore it functionally by reconstituting the pulp–dentin complex (Mao et al., 2012; He et al., 2019), but it seems difficult to achieve this goal with current clinical protocols (Kim et al., 2018). With the development of regenerative medicine using stem cells, cell-transplantation-based regeneration protocols for pulp regeneration have been steadily developed. The discovery and characterization of dental MSCs, especially DPSCs, raise expectations for pulp regeneration in RET in the future. Transplantation of DPSCs, which induce neuroangiogenesis, has achieved complete pulp regeneration in several studies (Murakami et al., 2015; Nakashima et al., 2017), with the crucial achievement of regeneration of neuroangiogenesis to achieve functional restoration of dental pulp. Nevertheless, it should be noted that current pulp regeneration is based only on autologous DPSCs. Transplantation with allogeneic DPSCs or pulp tissue from cryo-preserved cells or tissue banking clouds is an ideal clinical approach for pulp regeneration against infected pulp, especially in aged patients (Figure 5). Optimizing disinfection procedures as well as the application of proper scaffolds and/or factors for promoting neuroangiogenesis should also be key factors for allogeneic transplantation-induced pulp regeneration. Given the scientific evidence to date, cell-transplantation-based RET for pulp regeneration has been accepted as a promising treatment protocol. In addition, a functional pulp regeneration strategy through neurovascularization has the potential to become an innovative model for regenerative medicine and not only for dental pulp regeneration.
